# Diverse Roles of TRPV4 in Macrophages: A Need for Unbiased Profiling

**DOI:** 10.3389/fimmu.2021.828115

**Published:** 2022-01-20

**Authors:** Thanh-Nhan Nguyen, Ghizal Siddiqui, Nicholas A. Veldhuis, Daniel P. Poole

**Affiliations:** ^1^ Drug Discovery Biology Theme, Monash Institute of Pharmaceutical Sciences, Monash University, Parkville, VIC, Australia; ^2^ Australian Research Council (ARC) Centre of Excellence in Convergent Bio-Nano Science & Technology, Monash University, Parkville, VIC, Australia; ^3^ Drug Delivery, Disposition and Dynamics Theme, Monash Institute of Pharmaceutical Sciences, Monash University, Parkville, VIC, Australia

**Keywords:** TRP channels, mechanosensation, macrophage, inflammation, transient receptor potential vanilloid 4 (TRPV4)

## Abstract

Transient receptor potential vanilloid 4 (TRPV4) is a non-selective mechanosensitive ion channel expressed by various macrophage populations. Recent reports have characterized the role of TRPV4 in shaping the activity and phenotype of macrophages to influence the innate immune response to pathogen exposure and inflammation. TRPV4 has been studied extensively in the context of inflammation and inflammatory pain. Although TRPV4 activity has been generally described as pro-inflammatory, emerging evidence suggests a more complex role where this channel may also contribute to anti-inflammatory activities. However, detailed understanding of how TRPV4 may influence the initiation, maintenance, and resolution of inflammatory disease remains limited. This review highlights recent insights into the cellular processes through which TRPV4 contributes to pathological conditions and immune processes, with a focus on macrophage biology. The potential use of high-throughput and omics methods as an unbiased approach for studying the functional outcomes of TRPV4 activation is also discussed.

## Introduction

Inflammation is an essential defense mechanism generated by the immune system to protect the body from harmful stimuli or pathogen infection (1). Normally, inflammation is actively resolved to prevent tissue damage. This tightly regulated process involves the spatially and temporally controlled production of mediators leading to dilution of chemokine gradients to ensure that inflammatory responses subside in a timely fashion. Processes which resolve inflammation and rectify tissue homeostasis include reduction or cessation of tissue infiltration by neutrophils, apoptosis of spent neutrophils, down-regulation of chemokines and cytokines, macrophage transformation, and the initiation of healing (2, 3). Disruption of the control mechanisms that underlie these processes results in prolonged or uncontrolled inflammation, which is associated with chronic disease including inflammatory arthritis (4), inflammatory bowel disease (5), pulmonary diseases (6), atherosclerosis (7), foreign body response (8) and fibrosis (9).

The transient receptor potential (TRP) superfamily of ion channels plays important and emerging roles in inflammatory and immune-mediated diseases (10). One of the best characterized members is transient receptor potential vanilloid 4 (TRPV4), which is expressed by immune cells including macrophages (11–14), neutrophils (15), and dendritic cells. TRPV4 is a tetrameric ion channel with each subunit containing 6 transmembrane domains, a pore-forming loop, and 6 highly conserved ankyrin repeat domains in the cytoplasmic N-terminus (16). The functional role of TRPV4 and involvement in pathophysiology is most extensively defined for macrophages (12, 13, 17, 18). TRPV4 is expressed by various macrophage populations including tissue-resident macrophages located in the lung, gut, brain, liver, and skin (11–14, 19–21). Although TRPV4 has long been associated with pro-inflammatory roles, recent studies propose that TRPV4 activity can also influence macrophage function to promote the reduction or resolution of inflammatory damage (12, 13, 22). This raises a key question: *how can a single ion channel regulate two opposing processes?* In this review, we first highlight emerging evidence for the involvement of TRPV4 as a mechanosensitive channel in pathological conditions and immune responses, with a specific focus on macrophages. We then explore how the use of high-throughput omics approaches could reveal greater insight into the complex network of cellular pathways associated with TRPV4 activation.

## TRPV4: A Polymodal Ion Channel and Key Effector of Receptor Signaling

**Figure 1:**
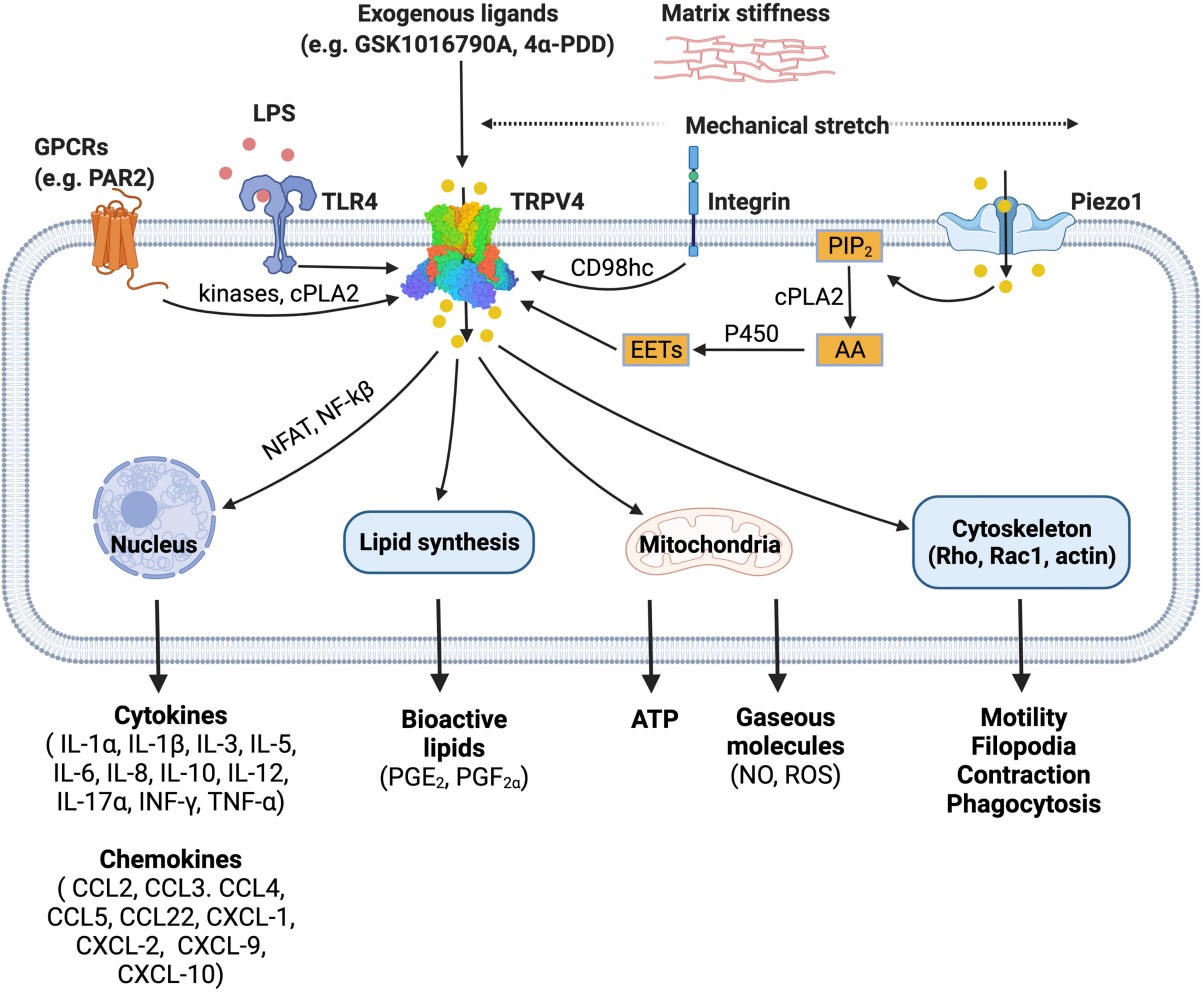
TRPV4 is a polymodal ion channel which can be activated directly or indirectly by a diverse range of stimuli including mechanical force, endogenous mediators, and pharmacological tools. TRPV4 signals through multiple pathways leading to a range of downstream effects on cellular function. The figure illustrates published activation pathways and associated outcomes. *TRPV4, transient receptor potential vanilloid 4; GPCRs, G protein-coupled receptors; TLR4, toll-like receptor 4; LPS, lipopolysaccharide; PIP_2_, phosphatidylinositol biphosphate; AA, arachidonic acid; cPLA2, cytosolic phospholipase A_2_; P450, cytochrome P450 epoxygenase; EETs, epoxyeicosatrienoic acids; NFAT, nuclear factor of activated T-cells; NF-kB, nuclear factor kappa B*. Figure created with BioRender.com.

TRPV4 was first identified as an osmosensitive channel due to its activation by hypotonicity (23, 24). It has since been shown to function as a polymodal non-selective cation channel that can be activated by a diverse array of stimuli including mechanical stress (25–28), warm temperature (above 27°C) (29, 30), endogenous polyunsaturated fatty acids (PUFAs) (31–33) and receptor-operated signaling (34). TRPV4 integrates cellular responses to these various stimuli, enabling this channel to influence a broad range of signaling and associated transcriptional events (11–14, 35–52), as summarized in **Figure 1**, and previously reviewed in detail (53).

TRPV4 is activated by hypoosmolarity, shear stress or direct deflection at cell-substrate contact points. Activation of TRPV4 by cellular indentation or membrane stretch is also commonly reported (25, 54), although the relative importance and generality of this mode of gating has recently been questioned based on electrophysiological studies (27, 28). This suggests that TRPV4 may only respond directly to specific mechanical cues. It is also evident that TRPV4 activation by hypotonic conditions and shear stress can indirectly modulate channel gating *via* production of lipid metabolites such as arachidonic acid and its metabolite 5′,6′-EET. This process requires PLA2 and cytochrome P450 epoxygenase activity (33, 55), suggesting that there are parallels between TRPV4 mechanosensitivity and its function as a receptor-operated channel. For example, G protein-coupled receptors (GPCRs) can also promote PLA2 and P450 activity to increase production of the same anandamide and arachidonic acid-derived lipid species. GPCRs, including members of the protease-activated, muscarinic, serotonin, and histamine receptor families, converge on TRPV4 through lipid signaling pathways, presumably as a mechanism to amplify specific signaling and transcriptional events (34). GPCR signaling can also sensitize and enhance the responsiveness of TRPV4 to these lipid metabolites by promoting direct phosphorylation of residues in its cytoplasmic N- and C-terminal domains by PKA, PKC and Src family tyrosine kinases (34). TRPV4 is proposed to be a key effector and ‘amplifier’ of sensory afferent nerve signaling. GPCR- and protein kinase-dependent sensitization of TRPV4 is associated with increased pain transmission and the peripheral release of neuropeptides and other mediators that promote neurogenic inflammation (56).

Recent studies have extended our understanding of how TRPV4 functions as an effector for receptor signaling and a broader integrator of mechanical cues in different cell types. Integrins are ubiquitously expressed transmembrane mechanoreceptors that are responsible for cell-cell interactions and cell adhesion (57). In endothelial cells, mechanical strain activates TRPV4-mediated Ca^2+^ influx *via* the β1 integrin-CD98hc axis, which is hypothesized to occur through a direct, physical interaction (58, 59). In this model, mechanical strain is sensed by β1 integrin, which initiates ultra-rapid signal transduction. The signal is transmitted from the cytoplasmic C terminus of β1 integrin to the N-terminal cytoplasmic ankyrin domain of TRPV4 *via* the transmembrane glycoprotein CD98hc, resulting in increased channel gating (59) (**Figure 1**).

Swain et al. have also recently demonstrated that TRPV4 is an effector protein for other ion channels (60, 61). Shear stress and mechanical pushing of pancreatic acinar cells indirectly activated TRPV4 *via* the fast-inactivating mechanosensitive ion channel Piezo1 (61). Piezo1 activation initiated a transient Ca^2+^ influx followed by a sustained elevation of intracellular Ca^2+^, an effect that was inhibited by the TRPV4 antagonist HC-067047 and mediated by PLA2 (60, 61).

The precise mechanisms involved in the TRPV4-dependent inflammatory response are not fully understood. However, it is speculated that changes to the extracellular matrix stiffness during inflammation can activate TRPV4. Scheraga et al. reported that TRPV4 is required for expression of dual-specificity phosphatase 1 (DUSP1) in response to LPS under pathophysiological matrix stiffness (>8kPa), but not under subthreshold matrix stiffness (1kPa) (12). DUSP1 is an inflammatory regulator, which inhibits c-Jun N-terminal kinases (JNK) and promotes p38 mitogen-activated protein kinases (MAPK) (12). In addition, calcium influx *via* TRPV4 also activates calcineurin which promotes nuclear factor of activated T-cells (NFAT) and nuclear factor kappa B (NF-kB) expression (62, 63). These studies illustrate an important role for TRPV4 in LPS-induced macrophage activation. Further detail outlining the involvement of TRPV4 in phenotypic switch by macrophages is provided in subsequent sections.

These established and emerging roles of TRPV4 as a key integrator and amplifier of mechano- and receptor-mediated signaling have been demonstrated for a range of distinct cell types including sensory neurons and endothelial cells. This is consistent with the generality of this function and highlights the associated challenges and opportunities when considering TRPV4 signaling and function as a potential therapeutic target.

## TRPV4-Expressing Macrophages as a Therapeutic Target for Resolving Inflammation

TRPV4 mRNA or protein has been detected in most organs and tissues (23, 24, 30, 31, 64–70) and is expressed by a broad range of cell types including neurons, urothelia, epithelia, immune cells, endothelial cells and aortic and airway smooth muscle (35, 65, 68, 71–73). This widespread expression pattern, coupled with multiple activating modalities, underlies the diverse roles of TRPV4 in physiological processes including volume- and osmo-sensing, thermoregulation, mechanosensation in the vasculature and urinary tract, cell barrier function, bone formation, metabolic disorders, pain, neurogenic inflammation, and gut motility (23, 29, 34, 53, 74–77). TRPV4 also performs critical pro-fibrotic roles and can detect and influence changes to the extracellular matrix (78). TRPV4 antagonists have been pursued and patented for several therapeutic applications including treatment or prevention of lung injury, heart failure, ischemic heart disease, and pain (79). Furthermore, pre-clinical and clinical studies have investigated TRPV4 inhibition as a therapeutic approach for treatment of osteoarthritis (80, 81), atherosclerosis (82), and cancer (83–85). More recently, the use of TRPV4 antagonists for managing comorbidities associated with SARS-CoV-2 infection such as lung edema has also been proposed (86). Despite these extensive efforts to define the importance of TRPV4 in cardiovascular, pulmonary, and inflammatory diseases, there is currently only one drug candidate (GSK2798745) approved for phase II clinical trials (87, 88). This clinical candidate is a small molecule, orally available inhibitor with low nanomolar potency (87).The apparent lack of therapeutic advancement may reflect limitations to our mechanistic understanding of the precise involvement of TRPV4 in inflammatory and protective processes.

In chronic disease, such as arthritis and joint pain, there are persistent changes to lipid production, osmolarity, increased presence of GPCR ligands (e.g., immune-derived peptides and proteases) and exposure to mechanical cues such as those associated with fibrosis. All these factors have the potential to promote sustained inflammatory signaling, edema, sprouting of nerve fibers, and angiogenesis, and most importantly, influence TRPV4 function (89). Studies using pharmacological tools and *trpv4^-/-^* mice have consistently shown that inhibition or loss of TRPV4 function reduces inflammatory processes and tissue edema. Accordingly, TRPV4 is often described as a pro-inflammatory mediator and a therapeutic target for treating inflammatory disease. However, most of these studies are acute in nature, and may not always adequately reflect proposed resolving roles for TRPV4. Macrophages are of particular interest for targeting inflammation and associated diseases. These cells orchestrate both inflammation and resolution, as summarized in **Table 1**, and recent evidence supports the dichotomous nature of TRPV4 in both homeostatic or protective roles and in pathophysiological pathways (12, 13). This includes roles in phagocytosis and cytokine production, both of which can be influenced by changes to the cellular environment in which the macrophages are located (11–13, 62).

**Table 1:** Summary of factors that are secreted in response to mechanical or pharmacological activation of TRPV4.

Secreted factors	Experimental conditions	Study models	Related conditions or physiological functions	Ref.
**↑** IL-1α, IL-1β, IL-6, IL-8 & CCL2	Stretch (cyclic 30%, 1.25 Hz)	M1 (GM-CSF induced) - hMDM	Lung injury	(44)
↑ TNF-α & CCL2	Stretch (cyclic 30%, 1.25 Hz)	M2 (M-CSF induced) - hMDM	Lung injury	(44)
↑ IL-1α, IL-6, IL-8 & CCL22	GSK101 (3 nM) or Stretch (cyclic 30%, 1.25 Hz)	NCI-H292	Lung injury	(44)
↑ IL-6 & CXCL1	Mechanical ventilation (30 ml/kg T_V_)	Balb/c mice (bronchoalveolar lavage fluid)	Lung injury	(44)
↓ IL-6, TNF-α & ROS	LPS (100 ng/mL)	TRPV4 siRNA RAW267.4	Lung injury	(62)
↑ NO & ROS	4α-PDD (10 μM)	mAM	Lung injury	(11)
↑ ROS	4α-PDD (10 μM)	Endothelial cells	Lung injury	(36)
↑ IL-6, CXCL1 & CXCL2	LPS (100 ng/mL)	*trpv4^-/-^* mBMDM	Pulmonary infection and injury in murine pneumonia model	(12)
	clinical strain of *Pseudomonas aeruginosa* embedded in agarose beads	*trpv4^-/-^* C57BL6 mice (whole lung lavage fluid)		
↑ IL-1β ↓ IL-10	LPS (100 ng/mL) & pathological matrix stiffness (25kPa)	*trpv4^-/-^* mBMDM	Pulmonary infection, injury, and fibrosis	(13)
↓ IL-1α, IL-1β, IL-3, IL-5, IL-6, IL-12p40, IL-12p70, IL-13, IL- 17α, INF-γ, TNF-α, CCL2, CCL3, CCL4, CCL5 & GM-CSF	LPS (50 mg/kg) + GSK219 (1 mg/kg)	C57BL6/J mice (blood concentration)	Sepsis	(45)
↑ IL-6, CCL2, CCL5 & CXCL1	Intracolonic administration of 4α-PDD (200 μg in 40% ethanol)	Mouse colonic tissue	Colitis	(35)
↑ IL-8, CCL2, CXCL9 & CXCL10	4α-PDD (100μM)	Caco-2	Colitis	(35)
↑ IL-8, CCL5 CXCL9 & CXCL10	4α-PDD (100μM)	T84	Colitis	(35)
↑ CCL2	GSK101 (10nM) or Hypotonic stimuli (200 mOsm/kg)	Muller glia	Acute retinal detachment	(46)
↑ Prostaglandin F_2α_	GSK101 (100 nM)	Aorta from high-salt diet-fed mouse	Hypertension	(43)
↑ Prostaglandin E2	GSK101 (300 nM)	mMM	GI motility	(14)
↑ ATP	GSK101 (100 nM) or Heat (25 -35.8°C)	Mouse esophageal keratinocytes	GERD, wound healing	(37, 47)
	GSK101 (100 nM) or 5,6-EET (500 nM) or mechanical stretch (120% lateral stretch)	RGE1-01	Gastric emptying	(48)
	GSK101 (0.01 mL, 50 nM)	Rat corneal epithelium + stroma, endothelium, cornea	Acute ocular hypertension	(49)
	GSK101 (10 nM – 10 μM)	Human bronchial epithelial cells	COPD (cigarette smoking-related)	(38)
	4α-PDD (3 μM, 10 μM)	HET-1A	Esophagitis and GERD	(39)
	GSK101 (100 nM)	Mouse cholangiocytes	Cholestatic liver disorders	(40)
	Stretch (400 μm/s) or 4α-PDD (10 μM)	Mouse urothelial cells	Bladder function	(41)
	4α-PDD (10 μM)	Astrocyte	N/A	(42)

mBMDM, mouse bone marrow-derived macrophages; hMDM, human blood monocyte-derived macrophages; mAM, mouse alveolar macrophages; mMM, mouse muscularis macrophages; GI, gastrointestinal; GERD, gastroesophageal reflux disease; COPD, chronic obstructive pulmonary disease; GSK101, GSK1016790A; GSK219, GSK2193874; 4α-PDD, 4α-Phorbol 12,13-didecanoate; N/A, not available. ↑ = increased; ↓ = decreased.

## TRPV4 Activity Influences Macrophage Polarization and Metabolism

### i) TRPV4 and Macrophage Polarization

Macrophages are a heterogeneous population of cells with the capability to change phenotype and perform specific roles in response to their microenvironment. For many years, the two main extremes of macrophage phenotype were widely accepted as M1 (so-called ‘classical’ pro-inflammatory phenotype) and M2 (‘non-classical’ anti-inflammatory phenotype). In reality, macrophages are highly versatile and the distinction between subsets is less clear. Metabolic reprogramming of macrophages is essential for phenotypic switch and immune responses. The M1 and M2 phenotypes have unique metabolic hallmarks (90–92). The manipulation of metabolic pathways in macrophages can alter their functions (93) and targeting of immunometabolism is a promising approach for blocking inflammatory signaling. For example, some anti-inflammatory drugs (e.g., dimethyl fumarate, metformin, methotrexate, and rapamycin) limit inflammation through targeting metabolic events in immune cells including macrophages (90).

TRPV4 activation is associated with phenotypic switch by macrophages (12, 13, 17, 51, 94). Current understanding of macrophage polarization is based largely on the use of biochemical cues such as cytokines or LPS to alter cellular phenotype. However, it is important to consider other biophysical factors originating from the microenvironment that may influence phenotype, such as exposure to shear stress and alterations in extracellular matrix stiffness. Several studies have explored how physical stimuli can affect macrophage phenotype, including the involvement of TRPV4 (see **Table 1**). Moderate cyclic stretch (7%, 0.8 Hz) applied to human peripheral blood mononuclear cells over a 7-day period increased the relative proportion of M2 cells (CD206^+^), whereas higher stretch (12%, 0.8 Hz) increased the M1-like (CCR7^+^) phenotype (95). In addition, cyclic or static stretch also triggered production of cytokines, chemokines, and enzymes by macrophages. This included expression of mRNA for iNOS, IL-6, MCP-1, and IL-10 (95, 96). Changes in stiffness of the surrounding extracellular matrix can affect surface protein expression and the secretion profile of macrophages. Previtera et al. (97) cultured murine BMDMs on 0.3–230 kPa polyacrylamide hydrogels and observed that macrophages grown on high stiffness substrates produced elevated levels of pro-inflammatory mediators relative to macrophages grown on softer substrates (97). However, a different study led by Chen et al. (98) showed that murine BMDMs cultured in polyacrylamide hydrogels at a low matrix stiffness (2.55 ± 0.32 kPa) displayed an M1-like phenotype, with enhanced CD86 cell surface expression and higher production of ROS, IL-1β and TNF-α. In contrast, a higher matrix stiffness (34.88 ± 4.22 kPa) drove the cells toward an M2-like phenotype with higher CD206 expression, and production of IL-4 and TGF-β (98). Direct comparison of these studies is complicated by the differences in the experimental design. In addition, although both used polyacrylamide hydrogels, Previtera et al. (97) pre-treated the gel with laminin (97), which has been shown to promote expression of pro-inflammatory factors in microglia (99) and reduce IL-10 secretion by THP-1 cells (100). However, these studies suggest that mechanosensitive receptors, such as TRPV4 (17), play a critical role in macrophage phenotypic switch in response to the biophysical properties of their environment. This is consistent with other non-TRPV4-related studies demonstrating that matrix stiffness has a profound influence on macrophage polarization states (100, 101) and warrants further investigation, as discussed by other manuscripts within this special issue.

### ii) TRPV4 and Macrophage Metabolism

Beyond expression of specific markers, macrophage phenotypes can also be differentiated based on their metabolic profiles, especially those associated with central carbon metabolism. Pro-inflammatory macrophages utilize glycolysis and the pentose phosphate pathway (PPP) to generate sufficient energy to meet higher ATP requirements. Fatty acid synthesis is increased, as this is required both as an energy production pathway and for synthesis of pro-inflammatory lipids, such as prostaglandins. At the same time, oxygen consumption is reduced, and the tricarboxylic acid (TCA) cycle and oxidative phosphorylation (OXPHOS) are suppressed. In contrast, macrophages with a protective phenotype have a normal TCA cycle and higher fatty acid oxidation rate (93).

Greater understanding of how TRPV4 influences macrophage phenotype at the metabolic level will provide further insight into the role of this channel in inflammation and inflammatory diseases. Although this has not been defined in detail, there is some evidence to suggest that TRPV4 can regulate central carbon metabolism, cellular respiration, and lipid metabolism. Several studies report that TRPV4 activation can increase production of reactive oxygen species (ROS) and nitric oxide (NO) (11, 36, 102) and evoke ATP release (37–42). In macrophages, ROS is largely generated through the NADPH oxidase pathway, while NO is mainly produced from arginine *via* the iNOS pathway. Both require NADPH as a co-factor. The high glycolytic flux of activated macrophages provides glucose-6-phosphate for the PPP, which is the main source of NADPH (93). Furthermore, the TRPV4 activator 4α-PDD reduces mitochondrial bioenergetics and oxygen consumption in pulmonary arterial endothelial cells after a 3 h incubation period (36). TRPV4 can also negatively regulate expression of peroxisome proliferator-activated receptor γ (PPARγ) coactivator 1α (PGC1α), mitochondrial uncoupling protein 1 (UCP1), and cellular respiration in adipocytes (103). PGC-1α is a transcriptional coregulator of pPPARγ, controlling the UCP1 promoter, which is involved in mitochondrial biogenesis and oxidative metabolism. PPARγ is an important transcription factor of M2 macrophages and is associated with fatty acid uptake and oxidation.

Pharmacological activation of TRPV4 also triggers secretion of pro-inflammatory lipid mediators including prostaglandins, suggesting a potential link between this ion channel and fatty acid biosynthesis (14, 43). Collectively, the metabolic profile of TRPV4-activated cells shares some similarities with the profile of the pro-inflammatory macrophage phenotype including high glycolysis, low OXPHOS activity and increased synthesis of pro-inflammatory lipids.

## Investigating Pleiotropic Roles of TRPV4 Using a Systems Biology Approach

This approach utilizes high-throughput omics platforms to interrogate complex biological systems. In contrast to most targeted studies outlined above, systems biology can comprehensively characterize molecular profiles at the level of the genome, transcriptome, proteome, peptidome, metabolome and lipidome in an unbiased manner (104–109). These approaches are well suited to the study of TRPV4 function in macrophages as these cells secrete mediators and their metabolic activity is highly regulated and linked to the inflammatory state.

### i) Metabolic Profiling in Mechanobiology and Immunology

Metabolomic methods have revealed novel biological pathways and important metabolites in inflammatory responses and have identified signature metabolites associated with different macrophage phenotypes (110–112). This includes the distinct metabolomic profiles of central carbon metabolism between M1 and M2 macrophages, as outlined above.

There are relatively few studies that examine the role of TRPV4 at a metabolic level (11, 36–42, 102). Furthermore, these are limited to targeted pathways including cellular respiration, NO production, and bioactive lipids, such as prostaglandins (**Table 1**). For example, targeted studies of isolated mouse alveolar macrophages have shown that 4-αPDD activates TRPV4 to promote Ca^2+^ influx and subsequent release of NO and superoxide (11). The combination of NO and superoxide can produce peroxynitrite, a strong oxidant involved in pathogen defense and inflammation (113, 114). Untargeted global profiling of TRPV4-induced macrophage phenotypes could help to address important questions of how and why TRPV4 can have both pro- and anti-inflammatory responses, and further understand the underlying mechanisms involved.

### ii) Profiling TRPV4-Mediated Lipid Synthesis and Metabolism

Macrophages are an important source of bioactive lipid mediators which are important determinants of the magnitude and duration of inflammatory signaling. In the onset phase of acute inflammation, eicosanoid lipid mediators (leukotrienes and prostaglandins) are released to promote inflammation (115, 116). At the resolution phase, cells switch to production of specialized pro-resolving mediators, such as lipoxins, resolvins, protectins, and maresins to resolve inflammation (115, 116). The imbalance of pro-inflammatory and pro-resolving mediators results in chronic inflammation (115, 116). Although TRPV4 activity can affect lipid metabolism in macrophages, including prostaglandin E2 (PGE2) production (14), this process has not been examined in detail and remains poorly characterized. The release of prostaglandins at the early stages of acute inflammation is important for a protective response. However, excessive production can promote chronic inflammation (115, 117, 118).

There is a clear need for more detailed investigation into how TRPV4 may influence lipid metabolism in the context of inflammatory disease. A comprehensive and unbiased lipidomics approach will provide mechanistic insight beyond that provided by current studies and significantly advance understanding of how TRPV4-mediated secretion of bioactive lipid mediators contributes to the initiation and resolution of inflammation.

### iii) Profiling the Protein Interactome

TRPV4 can directly or indirectly interact with a broad range of proteins (53, 56, 119). Mass spectrometry-based proteomics has become the core technology for large-scale investigation of protein-protein interactions with high confidence. Many purification methods have been developed to enable single protein complex characterization through to global interactome profiling (120). Commonly used workflows for purification of the target protein and its interactors include antibody-based affinity-purification mass spectrometry (AP-MS) (121), quantitative immunoprecipitation combined with knock-down (QUICK) (122), and proximity-ligation techniques such as BioID (123). The global interactome profiling requires biochemical techniques including fractionation by size-exclusion chromatography (SEC), ion-exchange chromatography (IEX), or perturbation co-behavior approach. The pros and cons of each of these workflows are covered elsewhere (120). Comprehensive analysis of the protein-protein interaction network will enable novel insight into the contribution of TRPV4 to cellular biology beyond what is possible with currently used methodology. Furthermore, this approach may facilitate identification of new avenues and targets to enable therapeutic modulation of TRPV4-dependent inflammatory signaling.

## Concluding Remarks

This review provides an overview of how TRPV4 influences macrophage function in pathological conditions and highlights the dual roles that this channel has in promoting and preventing inflammation. There is little doubt that TRPV4 is important for maintaining homeostasis and immune responses. This includes: 1) responses to pathogens and changes in biophysical factors including mechanical stress and matrix stiffness, 2) mediating inflammatory responses (phagocytosis, cytokine secretion) and balancing pro- and anti-inflammatory cytokine secretion, and 3) facilitating cell-cell communication *via* secreted factors. However, the underlying mechanisms involved in each role are not fully understood. In addition, cytokines and bioactive lipids secreted by macrophages are essential mediators of the inflammatory response. The importance of TRPV4 for macrophage polarization and associated production of cytokines is well documented. In contrast, current understanding of how TRPV4 regulates synthesis of bioactive lipids, protein expression, and protein-protein interactions is limited. This suggests that a focus on this specific research area using more comprehensive analysis methods is required. Application of high-throughput omics approaches to definitively characterize the effects of TRPV4 modulation on macrophages may reveal novel functions and pathways important for understanding the precise involvement of TRPV4 in inflammatory and protective processes. Similar methods have been widely applied in the immunology field, which has helped to further differentiate pro- and anti-inflammatory macrophage phenotypes (107, 124, 125). This information is critical for understanding how TRPV4 can influence both inflammatory and resolving processes and will contribute to future therapeutic targeting of TRPV4 in inflammatory diseases.

## Author Contributions

T-NN, GS, NV, and DP reviewed the literature and wrote the manuscript. All authors contributed to the article and approved the submitted version.

## Funding

ARC Centre of Excellence in Convergent Bio-Nano Science and Technology (CE14100036).

## Conflict of Interest

The authors declare that the research was conducted in the absence of any commercial or financial relationships that could be construed as a potential conflict of interest.

## Publisher’s Note

All claims expressed in this article are solely those of the authors and do not necessarily represent those of their affiliated organizations, or those of the publisher, the editors and the reviewers. Any product that may be evaluated in this article, or claim that may be made by its manufacturer, is not guaranteed or endorsed by the publisher.
